# Supplementary material for: Comprehensive analysis reveals a metabolic ten-gene signature in hepatocellular carcinoma
Source: PeerJ. 2020 May 26;8:e9201. doi: 10.7717/peerj.9201 (PMC7258935; doi:10.7717/peerj.9201)
Supplement: Supplemental Information 2 [file peerj-08-9201-s002.docx]

Table S2: Summary the Overlapping metabolism-related genes

| Type | Gene symbol |
| --- | --- |

| Overlapping metabolism-related genes | AACS GMPR2 CHPT1 PLCE1 AK1 ACSS3 PGM2 UGCG POLR2G PDE6A CYP26A1 XDH ECHS1 SDS INPP5K PDHA1 GSR GPX6 INPP5E NEU2 ACYP1 PFKP INPPL1 MGAM TK1 LIPF NUDT2 ACOT12 MTHFD2L GSTA1 GGT1 POLR2L SGPP2 ITPK1 PGP SULT1E1 TSTA3 UCKL1 IDO1 ADCY7 ACSS1 GLUD1 OPLAH PLA2G2C PTEN GFPT1 PLA2G3 HMOX1 APIP ME2 HMGCS2 HPD POLR1C GLO1 RENBP RDH10 P4HA2 NOS2 CA13 GYS1 PLA2G2F PFAS DCK ALOX12 PLCB2 BDH1 POLR3A SPTLC1 PNPT1 LALBA COX15 UROD GDA PDE1C LDHD UGT2B17 DNMT3L AGPAT1 PDE2A GALK1 CERK AK7 GOT1 AMY1C CKMT1B PNPLA3 BPNT1 ASL PLD2 GK MGLL AK3 AMY1A PIKFYVE UGT1A1 ALOX15B PI4KB ACADM MTMR1 PLCG2 PIK3C2B ALOX5 PCYT2 MAOB POLR3K FBP2 PTGDS NAMPT ENTPD6 NMNAT1 UGT1A9 PRIM1 CYP1A2 PRDX6 NUDT9 GK2 PLA2G12B CYP26C1 UROC1 ACADSB CYP2F1 SMPD4 AKR1B10 CMPK2 ACY1 INPP4B DGKG CYP2D6 GMPPB HGD ALLC GPX7 HNMT PNPLA4 ACYP2 NME2 PAPSS2 PFKFB2 ENTPD3 ACSM3 GSTM1 CDO1 UGDH CYP2C19 NOS1 CA14 ACSL3 CYP2S1 ACP5 CA4 PLCB1 PLCB4 PRPS1L1 ACACA LCLAT1 IMPDH2 PYGL CRLS1 PYGB CYP2U1 UGT2B7 GLUD2 OGDH DNMT1 DGUOK LYPLA1 ACAT1 GSS ALDOC NT5M PLA2G1B DGKI POLR1B HIBCH HMOX2 PLA2G5 DGKH GYS2 CES2 FMO3 AK4 PIK3CG ALDH3A2 PLCG1 GFPT2 POLR2K GUCY1A2 HAGHL IPPK BLVRA TAT MDH1 LCT NME7 CYP3A7 CD38 FMO4 PAFAH1B1 GBE1 PIP4K2A HCCS METTL6 BHMT LDHAL6B CYP4F2 GSTK1 SPTLC2 LPGAT1 PLA2G2A PCYT1A GSTA2 METTL2B SULT1A4 PIK3CA ALDH18A1 GALK2 TPI1 MAOA ETNK2 CBR3 POLR2F PLA2G2D CYB5R1 ALDH2 POLE2 ACSM2A AMY1B SGPL1 GMPR CYP2C18 GSTA4 POLE3 ALDH3A1 PLCD1 MGST3 FPGT GGT7 POLR1E PI4KA UGT1A7 PDE6C ACSM1 PLA2G15 POLR2B PSAT1 ACSM4 PNMT GNPDA1 ITPA UPB1 ADH5 AGPAT2 TRDMT1 UGT2B10 CDIPT ALDH4A1 UGT1A10 CMPK1 ENTPD4 UGT2B15 PAFAH2 ENTPD8 PMM1 NAT2 PLA2G4E MBOAT1 LCMT1 CKB GCLM CAD GNPNAT1 AGL ADI1 DTYMK MINPP1 P4HA3 PKLR ITPKB ACOX1 GMPPA ENPP7 QPRT HAO1 MTMR7 PDE6D PRODH GNE PIK3CB ACAT2 HADH NEU4 GNMT CDS1 GPD1 IDH2 GLUL DEGS1 PCK2 RRM2 LIPG HK2 G6PD FTMT RDH12 PIK3C3 CHST11 PRPS2 GCK POLR2I GUCY2C DNMT3B MGST2 EPHX1 NPR1 GPI ASS1 ALAD GSTM4 DAO PDE5A AWAT2 AGPAT3 POLR2J SULT1A3 PLCZ1 B4GALT1 CSAD CKMT1A IDO2 ABAT LPCAT4 ADCY5 GNPDA2 ALDH1A1 LDHB CYP1A1 MTAP DGAT2 NMNAT3 FMO5 PIP4K2B ACSL1 HPRT1 NME1-NME2 CMAS PLCB3 BDH2 TXNRD1 DPYD MIOX CYP3A43 ADK HMGCL SMPD3 CS TAZ PDE7A AKR1B1 LDHAL6A ACAA1 NUDT12 ACP2 HYI CA2 DEGS2 TPO UXS1 ACADL PAFAH1B2 AHCYL2 ASNS PTGES HAL MTHFD1L POLR1D GSTA3 PLD1 LDHC PC GUSB SORD PDHA2 DBH COX10 FAH SGMS2 DHRS4L2 GLDC ALOX15 AMDHD1 FBP1 MMAB PLA2G7 INPP5J ADH4 PAPSS1 PDE6B OCRL L2HGDH GLA NT5C OXCT2 CEL AGPS NEU1 DUT AKR1C2 PCCB ADCY6 DDC UGT2B4 NOS3 GSTP1 FADS2 NPR2 CHST12 ANPEP ADH1A NME5 CYP4F3 POLE4 ALDH1A2 CHIA MGST1 GSTM3 AHCY ASAH2 HADHB GGCT GUCY2F ASAH1 INMT ADSL KHK PDE10A RDH5 TREH FLAD1 BST1 AHCYL1 PIP4K2C NADSYN1 POLR2H CYB5R3 SMPD2 GPAM CANT1 AFMID MTR HK3 ALOX12B PDE4B MTMR2 GANC RDH8 ACO2 RRM1 POLR2A ODC1 DGKD TPMT CHST13 NMNAT2 PDE11A POLR3H PRIM2 SULT1A2 PIK3C2A CYP4A22 PGM2L1 SRR CHKB POLE GAA PPAT PTGES2 MAT1A ACER2 POLA1 CA3 PFKL CYP26B1 PNLIP DGKA PCCA GOT2 EARS2 OXCT1 NADK GALT POLD2 P4HA1 FMO2 FECH CYP2A6 TYR PDE9A CA8 DHRS4 POLR2J3 RPE65 UPP2 ARG2 CYP2C8 MTHFD1 GLS TXNRD2 AOC3 PLA2G6 MTHFD2 MTMR6 CYP3A5 NME6 ALDH1A3 FTH1 ZNRD1 HAO2 PAFAH1B3 ALDH6A1 G6PC2 ENTPD1 DHDH PFKFB1 SMPD1 TH UMPS ADCY3 GSTO2 ACMSD GALE NT5C2 ADH7 HAGH ACP1 HAAO DGKZ UPRT PDE8A AKR1C1 ADH1B SULT1A1 PLA2G4A PEMT DLD SARDH SYNJ1 PAICS LPL CA5B DGKE AKR1C4 NIT2 CAT POLR2J2 PLCD3 AMY2B LIPC CPOX ME3 GSTZ1 PGM1 WARS POLR3G MPI LPCAT2 NEU3 DCT GPD2 IMPA1 ASPA GCAT ITPKA PIP5K1A GSTO1 GPX5 AMY2A ACSL6 UGT1A8 AK5 ENPP1 CPS1 B4GALT6 CNDP1 UGT2B11 LDHA TYRP1 FHIT ENPP6 EPRS PPOX B4GALT2 POLR1A ACACB GBA3 GUK1 UGP2 CHAT ADCY4 DGAT1 KYNU CPT1B ACADVL SYNJ2 CES5A UPP1 RRM2B ADCY2 SPHK1 LAP3 SRM PHGDH IMPDH1 GNPAT OTC ACP6 TPH1 CA9 AMPD1 HEMK1 TBXAS1 CTPS2 ALDH1B1 CYP2B6 INPP1 HADHA AGK IDH1 PTGS2 PNLIPRP1 POLR2C ARG1 GPX3 NNT SEPHS1 GUCY2D PCK1 KMO SAT2 PRPS1 CBS GALC MIF HPGDS ADO ENTPD2 GAMT INPP5B ADH1C DGKB GSTA5 DPYS MARS CYP2C9 AGPAT4 HK1 DNMT3A NT5E G6PC WARS2 DDO PDE8B LPCAT3 HEXB NUDT5 GAD2 LTC4S CYP2A7 CA5A IL4I1 CYP2E1 PIK3C2G ADH6 AGXT INPP4A ACO1 PIP5K1B IMPA2 POLD1 GSTM5 MBOAT7 LCAT CTH PYCR1 GRHPR AMT ADSS UGT2A3 CHDH HMGCS1 TDO2 POLR3F SMS PFKFB3 ALAS2 AKR1C3 GPX4 MARS2 GMDS CPT1C GSTT2 IPMK TYMS DGKQ AMDHD2 GMPS GPT2 CPT1A ALDOB PFKFB4 PTGS1 GBA DCTD CYP2A13 SUCLG2 JMJD7-PLA2G4B UGT1A4 PLCD4 PDE4D ALDH3B1 GPX2 DLAT MAT2B POLD3 POLR3GL ALDH7A1 NT5C1B RDH16 AMPD2 TPH2 SEPHS2 CKMT2 PLA2G12A PYGM PDE3A ETNK1 PLA2G2E UGT1A6 PDE1B AKR1A1 ACSL5 DMGDH LYPLA2 PHPT1 FTCD GAL3ST1 MDH2 ACPP NME3 PLA2G4B PFKM SGMS1 GPX1 GART PYCR2 ENPP2 PIK3CD ADCY8 PHOSPHO1 ADSSL1 CDS2 POLR2E LRAT PMM2 ENOPH1 GLB1 NAGS AK2 CYP4A11 CA1 GPAT2 GSTM2 AGXT2 TXNDC12 PDHB UAP1 AGMAT NAGK LCMT2 ARSA ECI1 DHODH NAT1 SULT2B1 GGT6 ATIC RETSAT ACAA2 NPL FMO1 ME1 CDA POLR3C CA6 NANP PGS1 PGM3 PTDSS2 UGT8 GLYCTK CHIT1 GATM UROS KDSR UCK1 HEXA CHKA PGD NME1 CKM GAD1 MCEE POLR3D DHRS3 OGDHL CYP2J2 ALDOA HDC RDH11 LPCAT1 ADCY9 PCYT1B APRT ACHE PIP5K1C AOX1 GPD1L CP NT5C1A AANAT SUCLA2 PLA2G10 NANS SUOX ALAS1 GPT AADAT POLA2 AMD1 TRMT11 NNMT EHHADH UGT1A3 AMPD3 SHMT1 ALDH9A1 INPP5A ALDH3B2 CES1 ADCY10 PIPOX UGT1A5 ADA CA12 TYMP GGT5 SI MAT2A GCLC PDE1A CYP3A4 BAAT MBOAT2 ALDH5A1 PISD ENPP3 ACADS ACSL4 PRODH2 PDE4A POLD4 ECI2 SHMT2 EPHX2 MLYCD NME4 TK2 BLVRB GLS2 CYP1B1 CPT2 COMT AOC2 SAT1 SPHK2 POLR2D UGT2A1 ACOX3 GCDH ADCY1 PTGIS PAH UGT2B28 HMBS ACER3 DHRS9 ISYNA1 LTA4H PNLIPRP2 PDE7B PNP RFK ACY3 SCLY SGPP1 PDE3B UCK2 PTDSS1 CA7 CBR1 ACSM5 ENTPD5 PSPH ACSS2 POLR3B ACER1 SUCLG1 OAT MPST |
| --- | --- |
