# Supplementary material for: Comprehensive analysis reveals a metabolic ten-gene signature in hepatocellular carcinoma
Source: PeerJ. 2020 May 26;8:e9201. doi: 10.7717/peerj.9201 (PMC7258935; doi:10.7717/peerj.9201)
Supplement: Supplemental Information 3 [file peerj-08-9201-s003.docx]

Table S3: Summary the log FC and *P*-value for all the significantly metabolism-related DEGs

| Gene | | ConMean | | TreatMean | | LogFC | | PValue | | FDR | |
| --- | --- | --- | --- | --- | --- | --- | --- | --- | --- | --- | --- |
| **PGP** | 1.709225 | | 5.911624 | | 1.790212 | | 4.06E-14 | | 7.09E-13 | |  |
| **UGT2B11** | 0.212563 | | 11.80283 | | 5.795101 | | 4.22E-08 | | 1.29E-07 | |  |
| **GSTA4** | 2.629507 | | 8.505327 | | 1.693574 | | 1.12E-08 | | 3.81E-08 | |  |
| **PYCR1** | 0.290417 | | 6.450976 | | 4.473318 | | 0.000379 | | 0.000621 | |  |
| **CYP2C8** | 339.0208 | | 75.22395 | | -2.17211 | | 1.49E-14 | | 2.87E-13 | |  |
| **NME1** | 3.674193 | | 12.54866 | | 1.772034 | | 4.76E-14 | | 7.95E-13 | |  |
| **CNDP1** | 5.438884 | | 0.753609 | | -2.85142 | | 1.10E-13 | | 1.57E-12 | |  |
| **RDH16** | 90.57529 | | 27.13761 | | -1.73882 | | 4.02E-12 | | 2.94E-11 | |  |
| **GNPAT** | 4.728382 | | 15.54848 | | 1.717355 | | 2.17E-15 | | 7.25E-14 | |  |
| **POLD1** | 1.108935 | | 3.48863 | | 1.653485 | | 9.38E-16 | | 6.01E-14 | |  |
| **AKR1C2** | 8.038443 | | 31.33636 | | 1.96285 | | 0.000276 | | 0.000463 | |  |
| **DTYMK** | 1.904381 | | 7.133706 | | 1.905329 | | 3.19E-15 | | 9.82E-14 | |  |
| **PYGB** | 1.973518 | | 14.35311 | | 2.862522 | | 1.83E-15 | | 7.06E-14 | |  |
| **NMNAT2** | 0.055612 | | 0.294359 | | 2.404108 | | 6.85E-06 | | 1.46E-05 | |  |
| **PFKP** | 0.721207 | | 6.681861 | | 3.211765 | | 9.41E-05 | | 0.000167 | |  |
| **TK1** | 0.511909 | | 11.0754 | | 4.435326 | | 8.72E-16 | | 6.01E-14 | |  |
| **UGT2B7** | 204.2107 | | 71.62326 | | -1.51156 | | 5.04E-11 | | 2.69E-10 | |  |
| **NNMT** | 348.6525 | | 91.50199 | | -1.92991 | | 8.86E-11 | | 4.48E-10 | |  |
| **IMPDH2** | 6.820263 | | 23.87545 | | 1.807629 | | 4.96E-15 | | 1.27E-13 | |  |
| **ME1** | 1.763792 | | 7.073647 | | 2.003774 | | 0.007246 | | 0.00985 | |  |
| **ENTPD6** | 3.48628 | | 9.940478 | | 1.511627 | | 4.16E-12 | | 3.02E-11 | |  |
| **NAT2** | 20.00454 | | 3.069669 | | -2.70417 | | 4.92E-16 | | 5.40E-14 | |  |
| **GBA3** | 22.97669 | | 7.216443 | | -1.67081 | | 1.35E-11 | | 8.40E-11 | |  |
| **GYS2** | 29.57372 | | 8.966303 | | -1.72173 | | 5.30E-13 | | 5.74E-12 | |  |
| **PRIM1** | 1.056488 | | 3.013817 | | 1.512316 | | 1.12E-10 | | 5.62E-10 | |  |
| **CKMT2** | 0.116934 | | 1.667927 | | 3.834296 | | 0.038879 | | 0.048144 | |  |
| **EPRS** | 4.188074 | | 15.77791 | | 1.913548 | | 1.01E-14 | | 2.12E-13 | |  |
| **NOS2** | 0.157844 | | 0.48766 | | 1.627378 | | 6.33E-07 | | 1.58E-06 | |  |
| **PDE1C** | 0.081283 | | 0.281053 | | 1.789816 | | 1.48E-12 | | 1.29E-11 | |  |
| **GLUL** | 5.346244 | | 291.8155 | | 5.770387 | | 0.001764 | | 0.002614 | |  |
| **G6PD** | 0.337924 | | 7.748937 | | 4.519226 | | 3.60E-12 | | 2.69E-11 | |  |
| **TYRP1** | 0.045872 | | 0.250679 | | 2.450169 | | 0.000987 | | 0.001515 | |  |
| **OXCT1** | 0.337597 | | 1.290978 | | 1.935093 | | 0.018908 | | 0.024355 | |  |
| **CBR3** | 0.398794 | | 1.779306 | | 2.157598 | | 4.54E-09 | | 1.63E-08 | |  |
| **GLA** | 2.274602 | | 10.68262 | | 2.231579 | | 8.56E-15 | | 1.88E-13 | |  |
| **INMT** | 7.83896 | | 2.052266 | | -1.93344 | | 4.58E-12 | | 3.23E-11 | |  |
| **PLCD3** | 0.443646 | | 1.432432 | | 1.690985 | | 1.77E-10 | | 8.39E-10 | |  |
| **CA12** | 0.209002 | | 2.1582 | | 3.368238 | | 1.80E-05 | | 3.61E-05 | |  |
| **LCAT** | 52.29047 | | 10.64864 | | -2.29588 | | 1.26E-15 | | 6.31E-14 | |  |
| **CEL** | 0.134649 | | 0.593914 | | 2.141048 | | 1.24E-06 | | 2.92E-06 | |  |
| **PCK1** | 231.0468 | | 59.57464 | | -1.95541 | | 1.07E-12 | | 1.01E-11 | |  |
| **CKB** | 0.973691 | | 22.65804 | | 4.540416 | | 1.61E-05 | | 3.27E-05 | |  |
| **LPL** | 0.34576 | | 2.240318 | | 2.695862 | | 1.25E-14 | | 2.47E-13 | |  |
| **CYP1B1** | 0.819392 | | 13.84143 | | 4.078295 | | 0.029108 | | 0.036695 | |  |
| **SPHK1** | 0.601794 | | 2.245132 | | 1.899458 | | 0.003137 | | 0.004509 | |  |
| **ACSL4** | 3.476376 | | 32.78228 | | 3.23726 | | 2.02E-07 | | 5.60E-07 | |  |
| **ADH4** | 598.5762 | | 123.8354 | | -2.27311 | | 1.53E-13 | | 2.14E-12 | |  |
| **ALDOA** | 8.281004 | | 48.80772 | | 2.559232 | | 5.16E-11 | | 2.73E-10 | |  |
| **CYP2C9** | 210.2442 | | 61.57209 | | -1.77172 | | 1.47E-12 | | 1.29E-11 | |  |
| **ENTPD2** | 0.400256 | | 1.254293 | | 1.64788 | | 1.81E-06 | | 4.17E-06 | |  |
| **PLA2G1B** | 0.224546 | | 1.662456 | | 2.888233 | | 0.025268 | | 0.032244 | |  |
| **AGPAT1** | 5.243286 | | 15.16537 | | 1.532238 | | 1.92E-14 | | 3.61E-13 | |  |
| **LPCAT1** | 1.363468 | | 5.174915 | | 1.924254 | | 1.74E-08 | | 5.63E-08 | |  |
| **UROC1** | 31.13829 | | 10.02304 | | -1.63537 | | 4.29E-10 | | 1.88E-09 | |  |
| **TYMS** | 1.339413 | | 6.479642 | | 2.274313 | | 2.02E-10 | | 9.37E-10 | |  |
| **AKR1C3** | 4.827299 | | 59.50129 | | 3.623633 | | 1.02E-14 | | 2.12E-13 | |  |
| **ARG2** | 1.365279 | | 3.965872 | | 1.538442 | | 0.001045 | | 0.001601 | |  |
| **CYP4A22** | 45.66348 | | 15.84189 | | -1.5273 | | 3.67E-11 | | 2.03E-10 | |  |
| **PAFAH1B3** | 0.829216 | | 7.789553 | | 3.23172 | | 2.27E-13 | | 2.86E-12 | |  |
| **GAL3ST1** | 0.211847 | | 5.374263 | | 4.664969 | | 0.000344 | | 0.000568 | |  |
| **HAO2** | 65.40471 | | 17.61584 | | -1.89252 | | 1.14E-12 | | 1.07E-11 | |  |
| **TXNRD1** | 3.157999 | | 23.57983 | | 2.90047 | | 2.81E-11 | | 1.58E-10 | |  |
| **RDH8** | 0.033171 | | 0.206045 | | 2.634974 | | 0.00061 | | 0.000965 | |  |
| **DBH** | 9.053198 | | 1.446905 | | -2.64546 | | 3.77E-15 | | 1.07E-13 | |  |
| **CYP26A1** | 5.379982 | | 0.682035 | | -2.97968 | | 2.51E-14 | | 4.60E-13 | |  |
| **SRM** | 6.592463 | | 21.33142 | | 1.69409 | | 1.34E-12 | | 1.21E-11 | |  |
| **PHPT1** | 5.524823 | | 27.46507 | | 2.313598 | | 5.42E-13 | | 5.79E-12 | |  |
| **FLAD1** | 3.056939 | | 10.34589 | | 1.758898 | | 1.84E-17 | | 7.07E-15 | |  |
| **CHKA** | 2.562441 | | 8.044491 | | 1.650482 | | 1.41E-10 | | 6.91E-10 | |  |
| **GBA** | 2.771344 | | 16.56954 | | 2.579876 | | 1.14E-17 | | 7.07E-15 | |  |
| **CYP2B6** | 96.51407 | | 20.37439 | | -2.24398 | | 7.77E-12 | | 5.11E-11 | |  |
| **NT5M** | 0.457913 | | 1.761195 | | 1.94341 | | 1.52E-09 | | 5.84E-09 | |  |
| **LRAT** | 1.132606 | | 0.320793 | | -1.81993 | | 3.28E-14 | | 5.87E-13 | |  |
| **PSPH** | 1.286439 | | 5.960848 | | 2.212135 | | 4.28E-14 | | 7.31E-13 | |  |
| **BLVRA** | 2.202643 | | 7.268195 | | 1.722361 | | 1.63E-06 | | 3.81E-06 | |  |
| **ACSM1** | 2.43413 | | 10.45422 | | 2.102607 | | 0.000426 | | 0.000688 | |  |
| **ITPKA** | 0.251315 | | 2.643249 | | 3.394744 | | 1.54E-10 | | 7.51E-10 | |  |
| **ADH1A** | 282.3291 | | 89.97438 | | -1.64979 | | 1.56E-11 | | 9.53E-11 | |  |
| **AKR1B10** | 8.122238 | | 283.9571 | | 5.127652 | | 3.36E-07 | | 8.72E-07 | |  |
| **NEU1** | 3.913224 | | 17.85642 | | 2.190013 | | 6.69E-16 | | 6.01E-14 | |  |
| **FBP1** | 290.472 | | 83.65723 | | -1.79584 | | 5.84E-13 | | 6.15E-12 | |  |
| **UCK2** | 0.943953 | | 4.371643 | | 2.211389 | | 7.55E-17 | | 1.45E-14 | |  |
| **AADAT** | 9.120721 | | 2.871997 | | -1.66709 | | 8.59E-12 | | 5.60E-11 | |  |
| **LPGAT1** | 4.883516 | | 16.60752 | | 1.765845 | | 5.92E-11 | | 3.08E-10 | |  |
| **CYP4A11** | 173.8422 | | 56.66435 | | -1.61727 | | 1.69E-13 | | 2.33E-12 | |  |
| **POLR2K** | 5.777402 | | 19.67643 | | 1.767976 | | 3.02E-16 | | 4.64E-14 | |  |
| **CA4** | 0.073617 | | 0.716747 | | 3.283349 | | 4.05E-06 | | 8.97E-06 | |  |
| **SULT1A3** | 0.011134 | | 0.03261 | | 1.550286 | | 0.000266 | | 0.000448 | |  |
| **DHDH** | 0.154409 | | 0.569188 | | 1.88215 | | 1.95E-05 | | 3.83E-05 | |  |
| **CAD** | 1.159365 | | 3.387428 | | 1.546855 | | 5.88E-14 | | 9.22E-13 | |  |
| **P4HA2** | 1.399952 | | 4.171465 | | 1.575177 | | 2.55E-12 | | 2.02E-11 | |  |
| **RRM2** | 0.443665 | | 4.410921 | | 3.313538 | | 1.55E-15 | | 7.02E-14 | |  |
| **CKMT1A** | 0.050268 | | 0.150587 | | 1.582878 | | 0.000327 | | 0.000545 | |  |
| **MIOX** | 0.076449 | | 0.389171 | | 2.347828 | | 0.00098 | | 0.001507 | |  |
| **CYP1A2** | 123.6254 | | 30.52466 | | -2.01793 | | 1.81E-13 | | 2.41E-12 | |  |
| **AKR1C1** | 14.91924 | | 56.55261 | | 1.92242 | | 0.004172 | | 0.005874 | |  |
