# Supplementary material for: Comprehensive analysis reveals a metabolic ten-gene signature in hepatocellular carcinoma
Source: PeerJ. 2020 May 26;8:e9201. doi: 10.7717/peerj.9201 (PMC7258935; doi:10.7717/peerj.9201)
Supplement: Supplemental Information 4 [file peerj-08-9201-s004.docx]

Table S4: Summary the significantly metabolism-related genes associated with OS

| ID | | HR | HR.95L | HR.95H *P-*value | | FDR | |  |
| --- | --- | --- | --- | --- | --- | --- | --- | --- |
| **UCK2** | 1.176614 | | 1.124438 | 1.231211 | 2.10E-12 | | 1.52E-08 | |
| **TXNRD1** | 1.017929 | | 1.012387 | 1.023502 | 1.78E-10 | | 6.47E-07 | |
| **CAD** | 1.189158 | | 1.12515 | 1.256809 | 8.41E-10 | | 2.04E-06 | |
| **G6PD** | 1.015744 | | 1.010545 | 1.02097 | 2.42E-09 | | 4.41E-06 | |
| **RRM2** | 1.066549 | | 1.039212 | 1.094605 | 1.15E-06 | | 1.68E-03 | |
| **ME1** | 1.039295 | | 1.021651 | 1.057244 | 1.02E-05 | | 1.24E-02 | |
| **DTYMK** | 1.045269 | | 1.024742 | 1.066208 | 1.21E-05 | | 1.26E-02 | |
| **LPCAT1** | 1.042391 | | 1.022911 | 1.062242 | 1.61E-05 | | 1.46E-02 | |
| **ENTPD2** | 1.11098 | | 1.058084 | 1.166521 | 2.35E-05 | | 1.90E-02 | |
| **POLD1** | 1.134208 | | 1.069078 | 1.203307 | 3.00E-05 | | 2.18E-02 | |
| **PRIM1** | 1.142842 | | 1.071774 | 1.218623 | 4.58E-05 | | 3.03E-02 | |
| **CYP2C9** | 0.99574 | | 0.993435 | 0.998051 | 0.000307 | | 1.86E-01 | |
| **GLA** | 1.033818 | | 1.014542 | 1.053461 | 0.000533 | | 2.99E-01 | |
| **SRM** | 1.012853 | | 1.005402 | 1.020359 | 0.000699 | | 3.63E-01 | |
| **AKR1B10** | 1.000635 | | 1.000266 | 1.001004 | 0.000746 | | 3.62E-01 | |
| **TYMS** | 1.036586 | | 1.014855 | 1.058783 | 0.000887 | | 4.03E-01 | |
| **AKR1C3** | 1.00417 | | 1.001673 | 1.006674 | 0.001052 | | 4.50E-01 | |
| **LCAT** | 0.976318 | | 0.96168 | 0.991179 | 0.001874 | | 7.57E-01 | |
| **ADH4** | 0.998478 | | 0.997519 | 0.999437 | 0.001884 | | 7.21E-01 | |
| **FLAD1** | 1.039165 | | 1.013355 | 1.065633 | 0.002755 | | 1.00E+00 | |
| **PYCR1** | 1.022068 | | 1.007566 | 1.036779 | 0.002756 | | 9.55E-01 | |
| **PSPH** | 1.03369 | | 1.011036 | 1.056853 | 0.003382 | | 1.12E+00 | |
| **ADH1A** | 0.99749 | | 0.995786 | 0.999197 | 0.003962 | | 1.25E+00 | |
| **TK1** | 1.01406 | | 1.004262 | 1.023954 | 0.004824 | | 1.46E+00 | |
| **ALDOA** | 1.003105 | | 1.000929 | 1.005287 | 0.005152 | | 1.50E+00 | |
| **GBA** | 1.022596 | | 1.006431 | 1.03902 | 0.005988 | | 1.68E+00 | |
| **AKR1C1** | 1.002962 | | 1.000804 | 1.005125 | 0.007121 | | 1.92E+00 | |
| **EPRS** | 1.026315 | | 1.006112 | 1.046924 | 0.010447 | | 2.71E+00 | |
| **DHDH** | 1.181411 | | 1.037437 | 1.345365 | 0.011928 | | 2.99E+00 | |
| **GYS2** | 0.979315 | | 0.963032 | 0.995875 | 0.014559 | | 3.53E+00 | |
| **NME1** | 1.014217 | | 1.002372 | 1.026202 | 0.018513 | | 4.35E+00 | |
| **PCK1** | 0.99767 | | 0.995554 | 0.99979 | 0.031264 | | 7.11E+00 | |
| **PLA2G1B** | 1.020427 | | 1.001635 | 1.039571 | 0.03299 | | 7.27E+00 | |
| **RDH16** | 0.994572 | | 0.989531 | 0.99964 | 0.035815 | | 7.66E+00 | |
| **PFKP** | 1.010538 | | 1.000595 | 1.020579 | 0.037724 | | 7.84E+00 | |
| **RDH8** | 1.207356 | | 1.009633 | 1.4438 | 0.038921 | | 7.87E+00 | |
| **ENTPD6** | 1.03036 | | 1.000223 | 1.061407 | 0.048308 | | 9.50E+00 | |
|  |  | |  |  |  | |  | |
