# Supplementary figures and images for: Comprehensive analysis reveals a metabolic ten-gene signature in hepatocellular carcinoma
Source: PeerJ. 2020 May 26;8:e9201. doi: 10.7717/peerj.9201 (PMC7258935; doi:10.7717/peerj.9201)

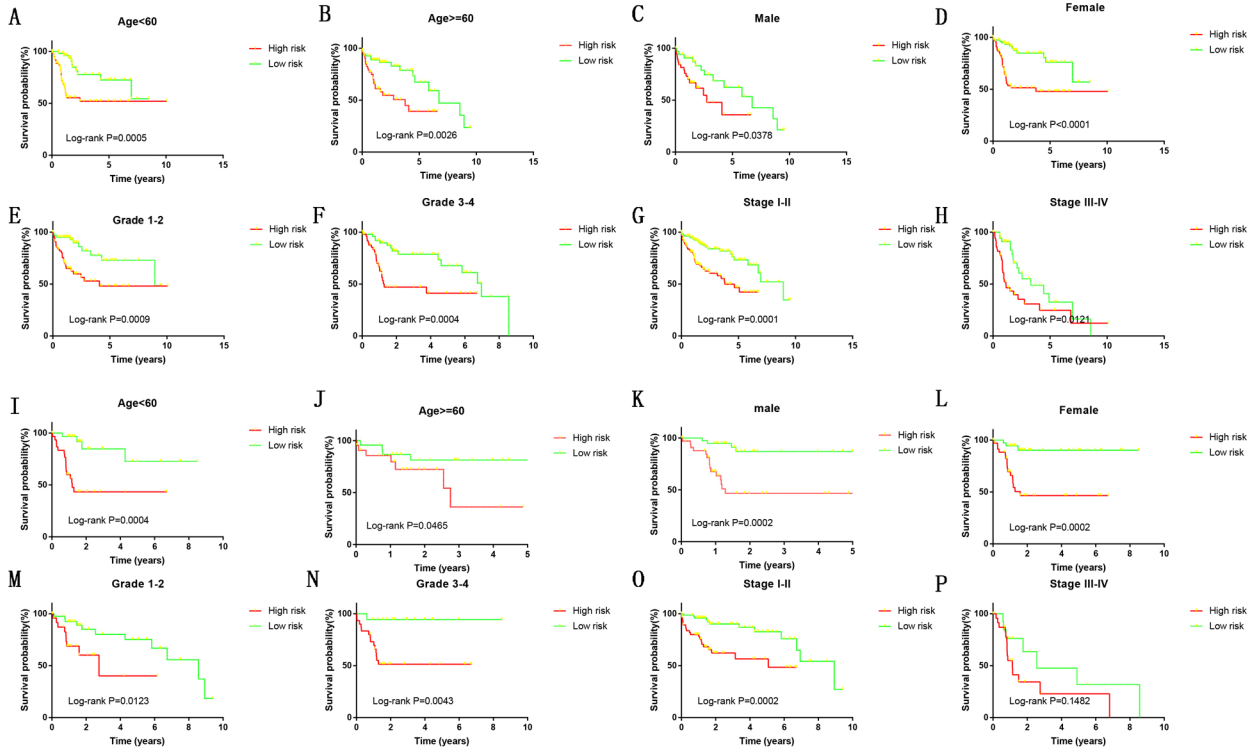

Supplement: Supplemental Information 5 — (A-B) Age <60 and >=60 in training cohort (C-D) Male and female in training cohort (E-F) Grade 1-2 and grade 3-4 in training cohort (G-H) Stage I-II and stage III-IV in training cohort (I-J) Age <60 and >=60 in internal testing cohort (K-L) Male and female in internal testing cohort (M-N) Grade 1-2 and grade 3-4 in internal testing cohort (O-P) Stage I-II and stage III-IV in internal testing cohort [file peerj-08-9201-s005.pdf]

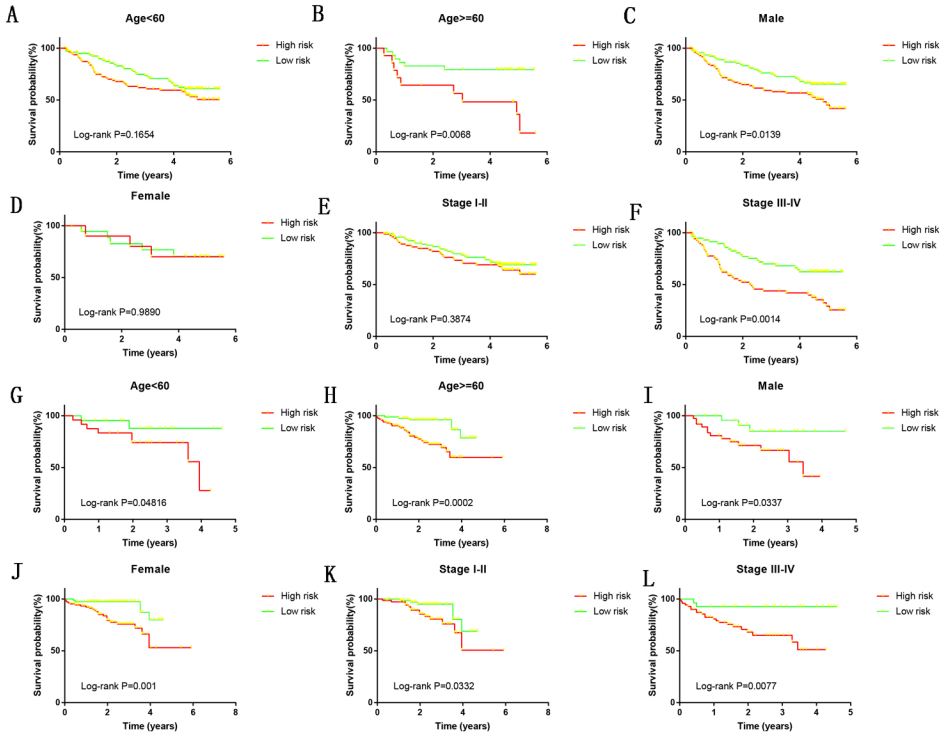

Supplement: Supplemental Information 6 — (A-B) Age <60 and >=60 in GSE14520 testing cohort (C-D) Male and female in GSE14520 testing cohort (E-F) Stage I-II and stage III-IV in GSE14520 testing cohort (G-H) Age <60 and >=60 in ICGC testing cohort (I-J) Male and female in ICGC testing cohort (K-L) Stage I-II and stage III-IV in ICGC testing cohort [file peerj-08-9201-s006.pdf]

**A****Age<60**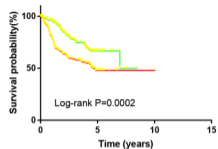**B****Age>=60**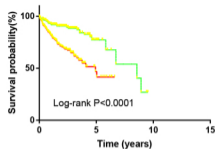**C****Male**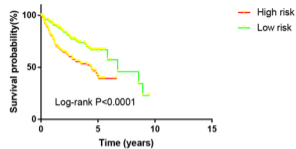**D**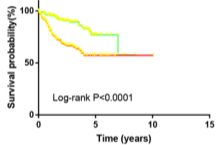**E****Stage I-II**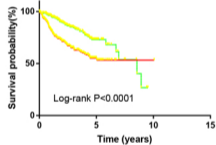**F****Stage III-IV**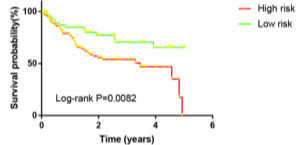**G****Stage I**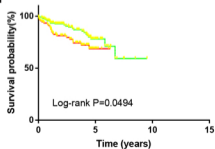**H****Stage II**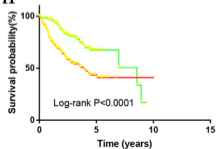**I****Stage III**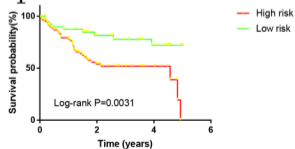

Supplement: Supplemental Information 7 — (A-B) Age <60 and >=60 in entire testing cohort (C-D) Male and female in entire testing cohort (E-F) Stage I-II and stage III-IV in entire testing cohort (G-I) Stage I, stage II and stage III in entire testing cohort [file peerj-08-9201-s007.pdf]

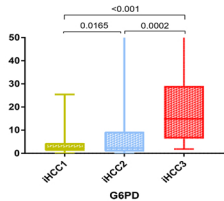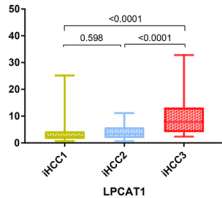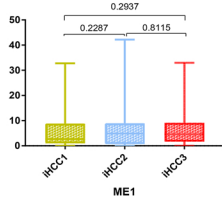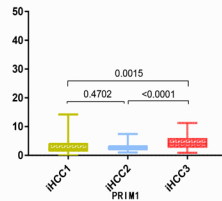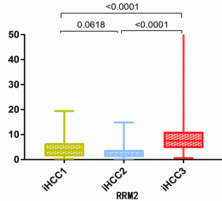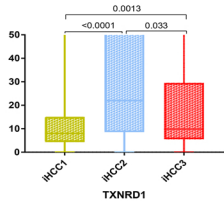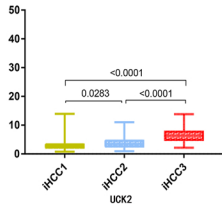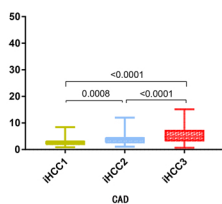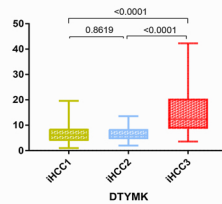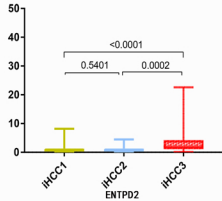

Supplement: Supplemental Information 8 [file peerj-08-9201-s008.pdf]

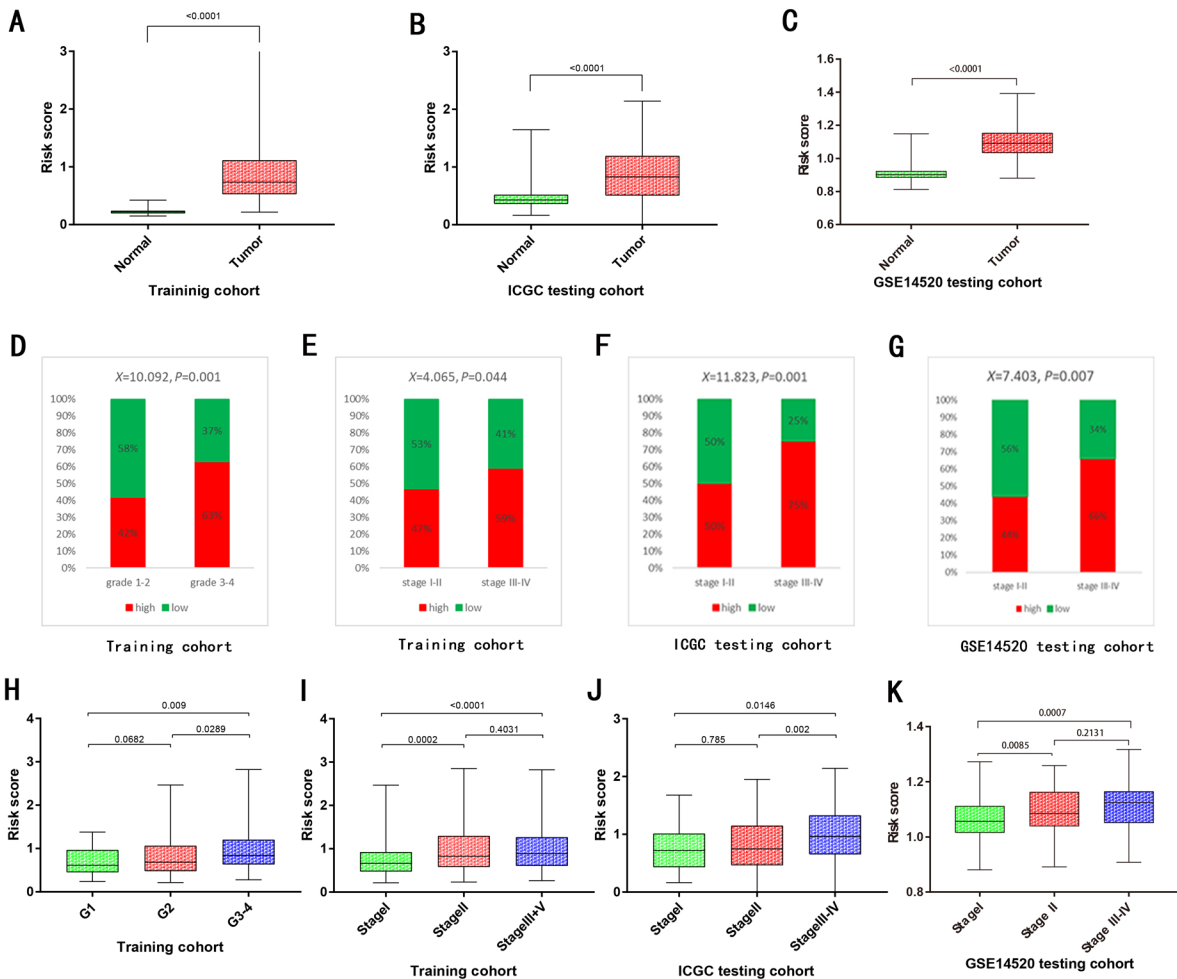

Supplement: Supplemental Information 9 — The risk score was grouped by (A-C) Tissue type (H) Tumor grade (I-K) TNM stage. The distribution of high-risk and low-risk patients in different stages and grades (D) Tumor grade (E-G) TNM stage A, D, E, H, I were from training cohort. B, F, J were from ICGC testing cohort. C, G, K were from GSE14520 testing cohort [file peerj-08-9201-s009.pdf]

**A**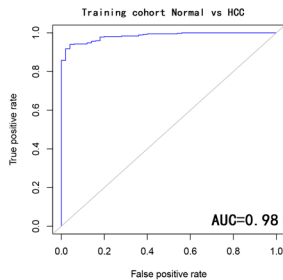**B**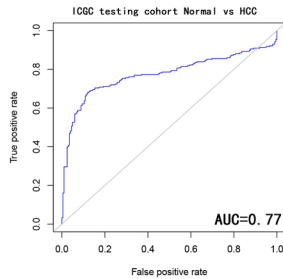**C**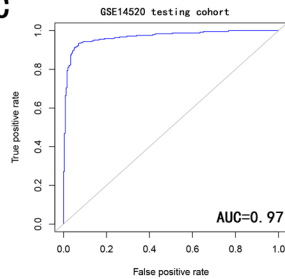**D**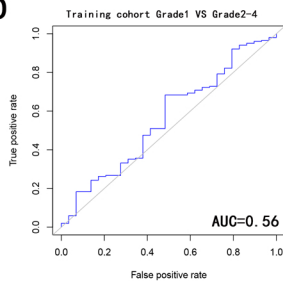**E**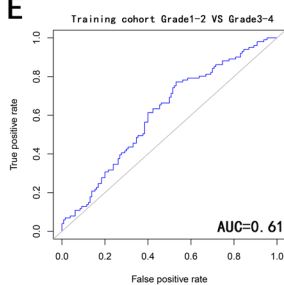**F**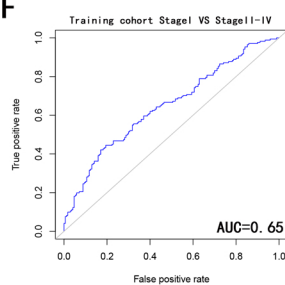**G**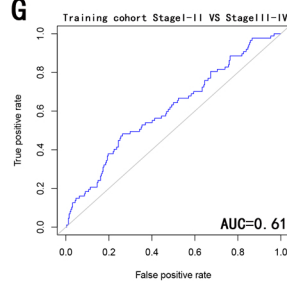**H**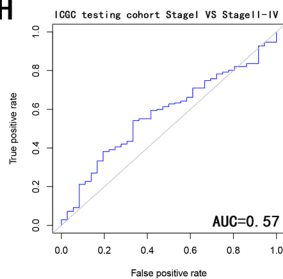**I**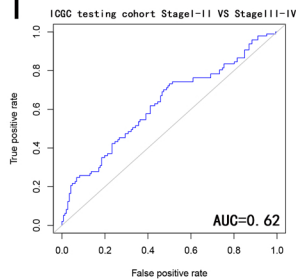**J**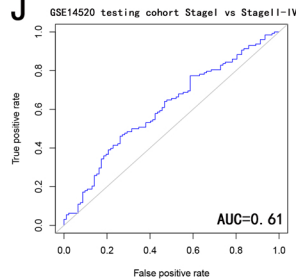**K**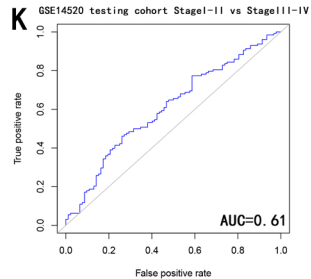

Supplement: Supplemental Information 10 — The capacity in differentiating between normal and HCC(A-C), different grade (D-E), different stage ( F-K). A, D, E, F, G were from training cohort. B,H,I were from ICGC testing cohort. C,J,K were from GSE14520 testing cohort [file peerj-08-9201-s010.pdf]

A

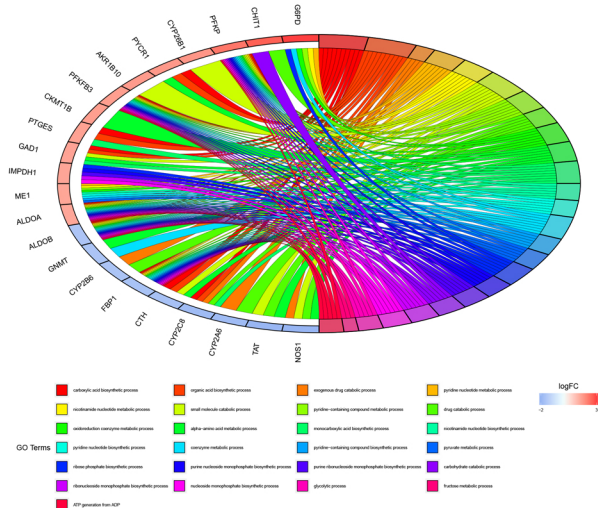

B

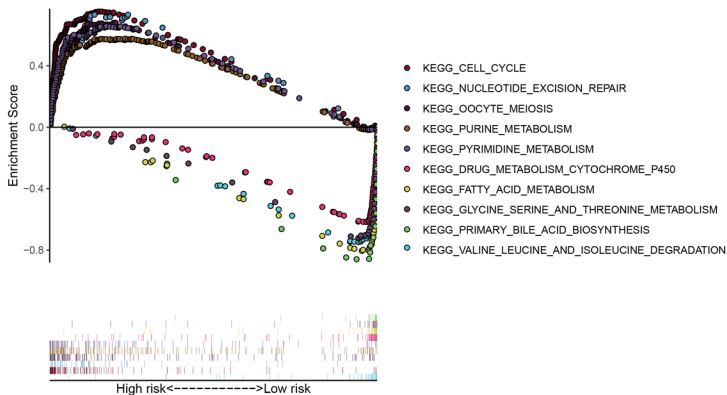

Supplement: Supplemental Information 11 — (A) Biological processes (B) KEGG signaling pathway [file peerj-08-9201-s011.pdf]
